# Targeting the gut‒kidney axis for lupus nephritis treatment: multimechanism regulatory strategies and evidence from Traditional Chinese medicine

**DOI:** 10.1186/s13020-026-01426-9

**Published:** 2026-05-27

**Authors:** Qingyun Fang, Jiabao Liu, Chen Xuan, Chaofan Li, Xin Jiang, Shuangna Zhang, Qinggang Li, Xiaomin Liu, Qun Liu, Li Zhang, Yong Wang, Jing Cui, Yilun Qu, Jie Zhang, Ping Li, Xiangmei Chen

**Affiliations:** 1 School of Basic Medical Sciences, Chengdu University of Traditional Chinese Medicine, Chengdu, 610075 China; 2 Department of Nephrology, First Medical Center of Chinese PLA General Hospital, State Key Laboratory of Kidney Diseases, National Clinical Research Center for Kidney Diseases, Beijing Key Laboratory of Medical Devices and Integrated Traditional Chinese and Western Drug Development for Severe Kidney Diseases，Beijing Key Laboratory of Digital Intelligent TCM for the Preventionand Treatment of Pan-vascular Diseases，Key Disciplines of National Administration of Traditional Chinese Medicine(zyyzdxk-2023310), Beijing, 100853 China; 3 School of Medicine, Nankai University, Tianjin, 300071 China

**Keywords:** Lupus nephritis, Gut–kidney axis, Traditional Chinese medicine, Gut microbiota

## Abstract

Lupus nephritis (LN) treatment remains challenging because of the limited efficacy and substantial side effects of conventional immunosuppressive therapies. Traditional Chinese medicine (TCM), with its holistic and multitarget approach, offers unique therapeutic potential. The emerging gut–kidney axis theory provides a new framework for understanding LN pathogenesis by linking gut dysbiosis and intestinal barrier injury to renal inflammation. This review systematically examines the role of gut–kidney axis dysregulation in LN progression and establishes connections between the TCM spleen–kidney correlation theory and this modern concept. Accumulating evidence suggests that TCM compounds and active ingredients alleviate renal injury and improve LN through multiple mechanisms. TCM compounds modulate the gut microbiota composition, enhance intestinal barrier integrity, reduce endotoxin translocation, and suppress systemic inflammation. These findings position the gut–kidney axis as a critical target for TCM intervention. Through multicomponent synergy, TCM restores gut homeostasis and inhibits aberrant immune responses. Future studies should integrate multiomics approaches, including metagenomics and metabolomics, and prospective clinical trials should dynamically track the gut microbiota and metabolite profiles in LN patients. Such investigations will clarify the precise mechanisms by which TCM modulates the gut–kidney axis and facilitate the development of personalized TCM-based therapeutic strategies.

**Graphical abstract:**

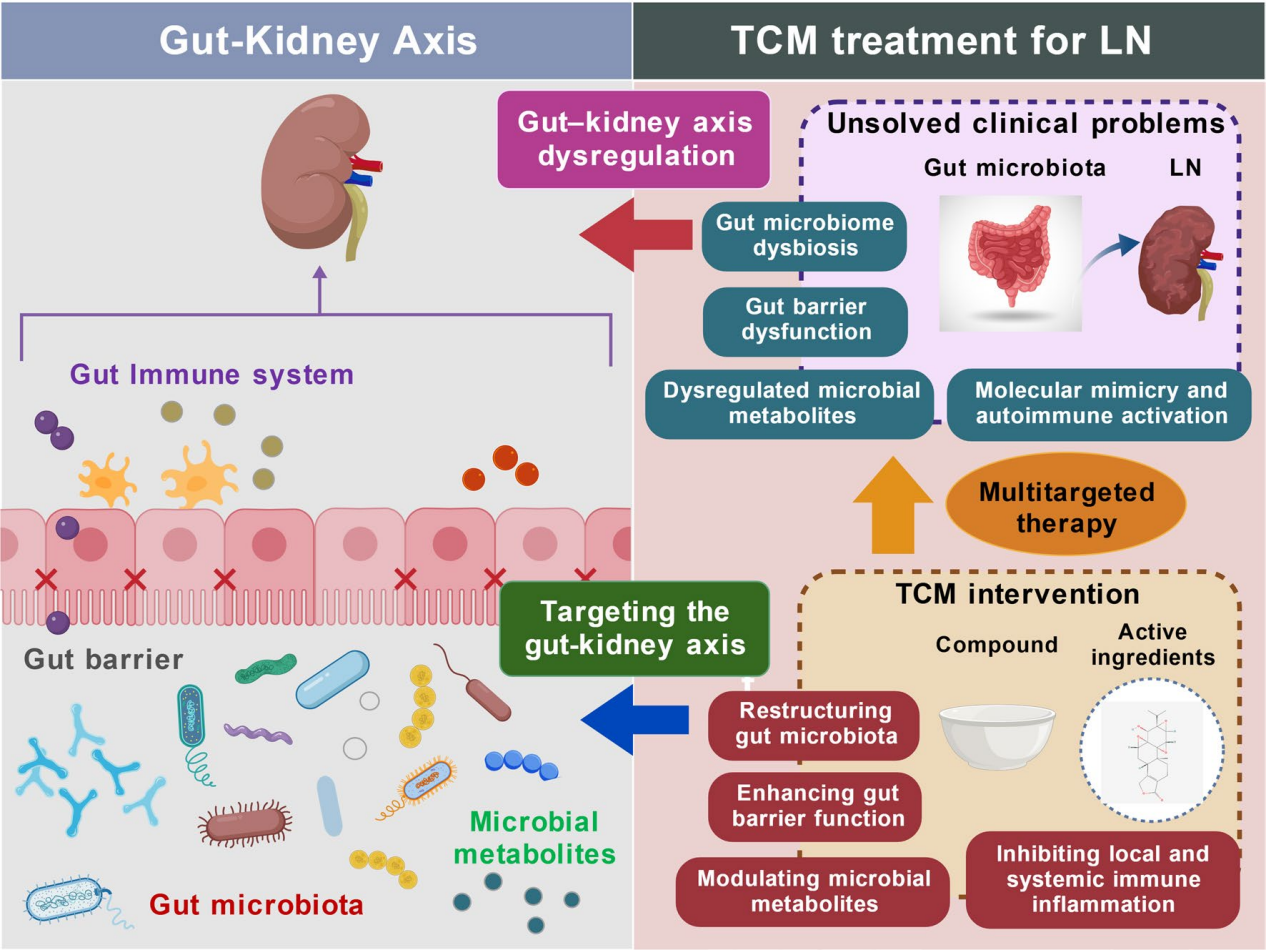

## Background

Lupus nephritis (LN) remains among the most severe and common complications of systemic lupus erythematosus (SLE), affecting more than 50% of SLE patients and significantly increasing the risk of end-stage renal disease and mortality [1–3]. Conventional immunosuppressive therapies, including cyclophosphamide and mycophenolate mofetil, provide only partial disease control, and their long-term use is limited by heterogeneous treatment responses, high recurrence rates, and significant risks of infection and metabolic toxicity [2]. Thus, safer and more precise therapies targeting core LN mechanisms are urgently needed.

The recently proposed gut–kidney axis theory offers a new perspective on how systemic autoimmunity affects the kidneys [4–6]. This theory posits that gut dysbiosis and impaired intestinal barrier function facilitate the translocation of microbial products, such as Lipopolysaccharide (LPS), into circulation. These products activate innate immunity via Toll-like receptor pathways, driving aberrant adaptive autoimmune responses and ultimately leading to immune-mediated renal injury [7, 8]. LN patients commonly exhibit gut microbiota disturbances and increased intestinal permeability, a condition termed “leaky gut”, which aligns with this framework [9–11]. The gut is therefore considered a key upstream regulator of systemic immune dysregulation and LN progression, providing a rationale for microbiota-targeted interventions.

Traditional Chinese medicine (TCM) has accumulated extensive clinical experience in treating LN [12]. The holistic principles and syndrome differentiation in TCM align closely with the cross-organ regulation embodied in the gut–kidney axis. Specifically, the TCM spleen–kidney correlation theory parallels multiple pathophysiological aspects of this axis. However, the abstract nature of TCM theories and the limited understanding of their molecular mechanisms have hindered scientific validation and clinical translation in modern medicine.

**Fig. 1:**
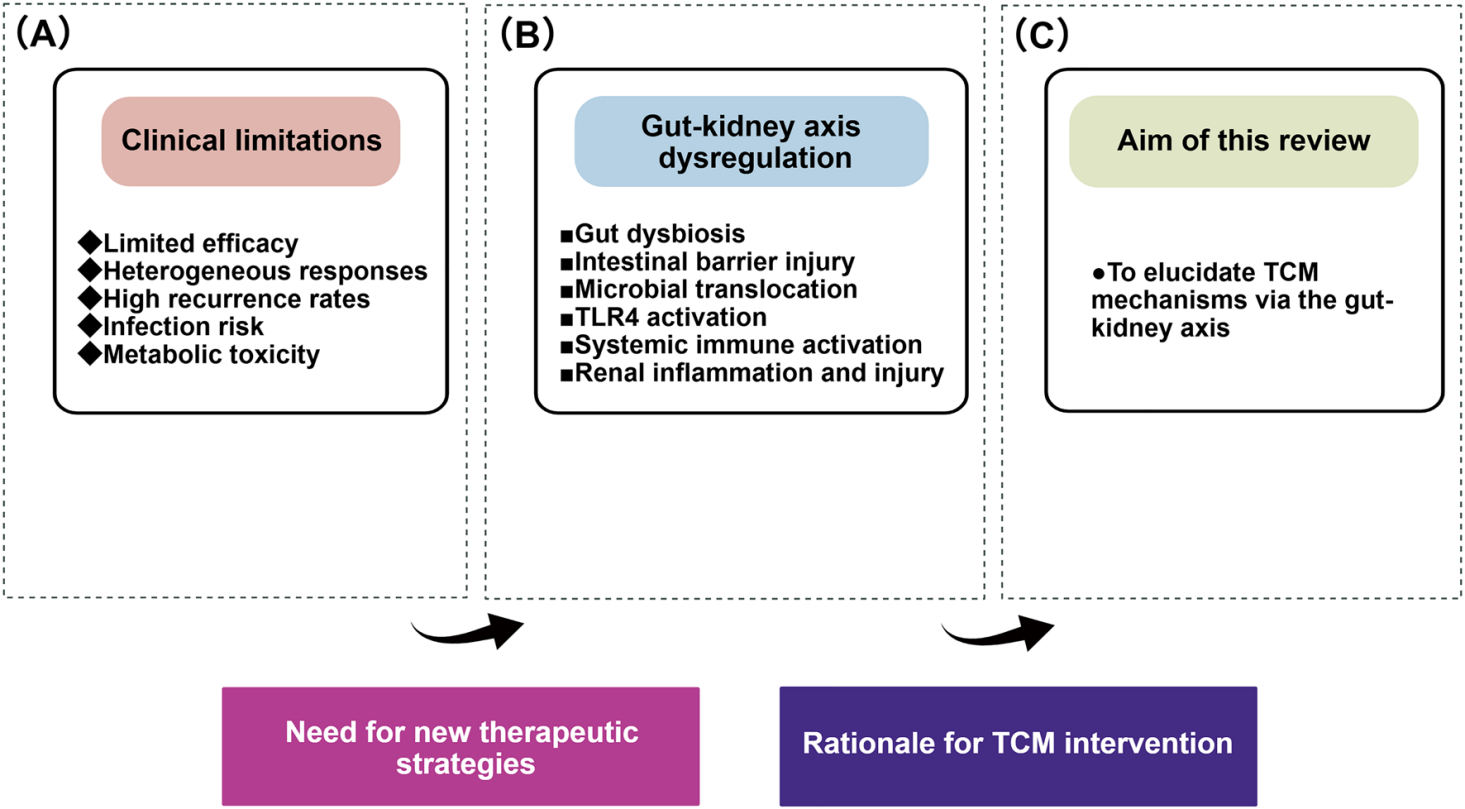
Rationale for targeting the gut–kidney axis in LN therapy. **A** Clinical limitations of conventional immunosuppressive treatments for LN, including limited efficacy, heterogeneous responses, high recurrence rates, and risks of infection and metabolic toxicity. **B** Gut–kidney axis dysregulation in LN: gut dysbiosis and intestinal barrier injury promote the translocation of microbial products into circulation, triggering systemic immune activation via TLR4 signaling and ultimately driving renal inflammation and injury. **C** Aim of this review: to elucidate the modern mechanistic rationale for TCM-based LN treatment within the framework of the gut–kidney axis theory. This figure was created with BioGDP [13]

This review elucidates the mechanistic basis for TCM treatment of LN through the lens of the gut–kidney axis. We first review the role of this axis in LN pathogenesis, and then examine TCM mechanisms within this framework. We systematically review TCM formulas and active components experimentally demonstrated to improve LN by modulating the gut–kidney axis, and discuss candidate agents with therapeutic potential. Finally, we consider current challenges and propose that future studies integrating cutting-edge technologies will clarify the molecular mechanisms through which TCM regulates this axis, ultimately translating TCM concepts into precisely targeted therapeutic strategies (Fig. 1).

**Fig. 2:**
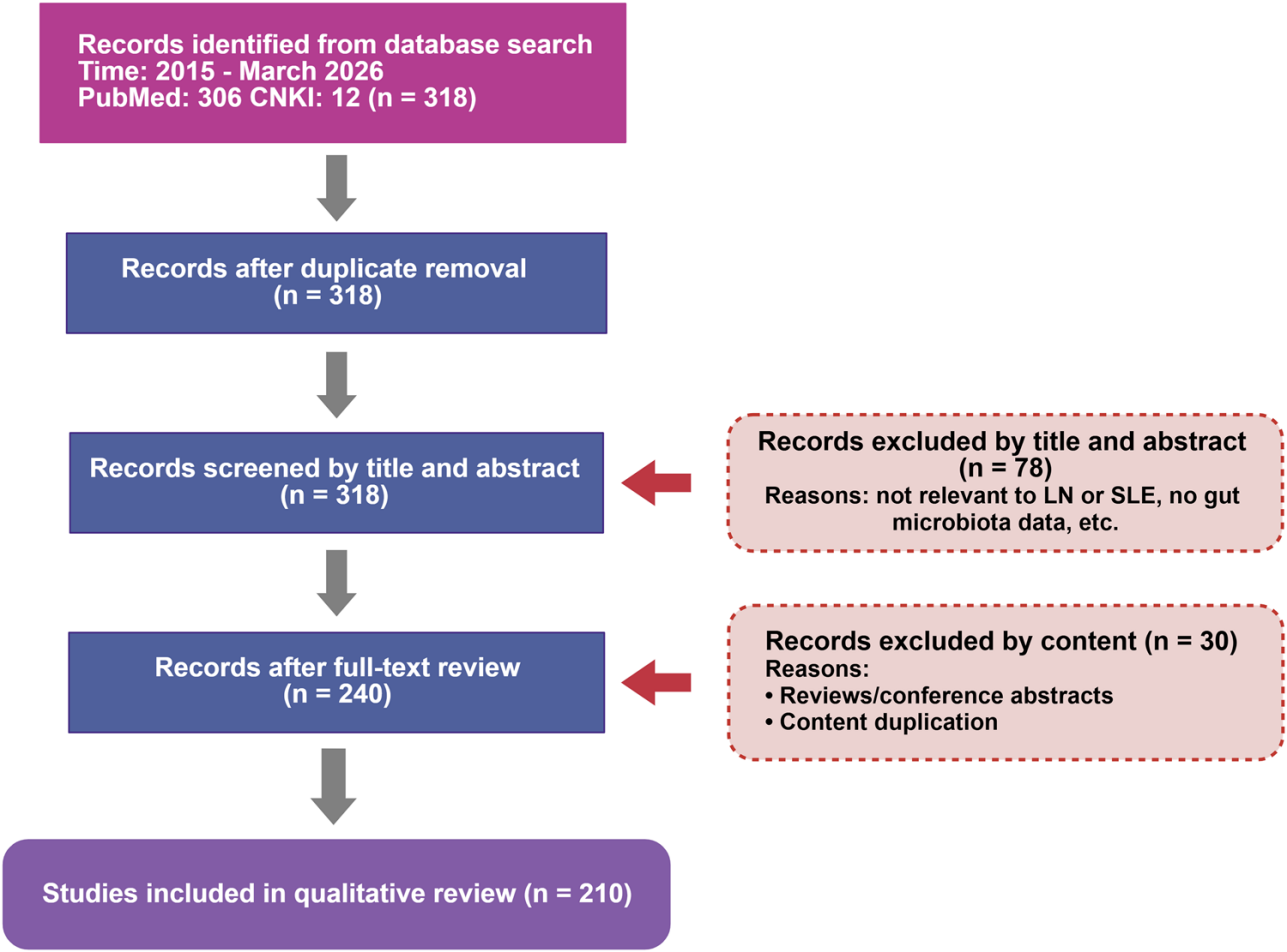
The flow diagram of the literature search and screening process. A total of 318 records were identified from PubMed (n = 306) and CNKI (n = 12). After title, abstract, and full-text screening, 210 studies were included in this review

We systematically searched PubMed and CNKI for publications from January 2015 to March 2026 using various keyword combinations including “lupus nephritis,” “systemic lupus erythematosus,” “gut microbiota,” “gut–kidney axis,” “intestinal barrier,” and “dysbiosis.” The search yielded 318 records (PubMed: 306; CNKI: 12). After removing duplicates and excluding conference abstracts and case reports, all 318 records remained. The title and abstract screening excluded 78 records, and the full-text review excluded an additional 30 records, resulting in 210 studies for inclusion in this review (Fig. 2).

The inclusion criteria were as follows: (1) studies related to the gut–kidney axis, gut microbiota, or intestinal barrier in LN or SLE; (2) studies on TCM interventions with outcomes related to the gut microbiota, intestinal barrier, or renal function; and (3) original research or review articles in peer-reviewed journals. Conference abstracts, case reports, and non-English/Chinese articles were excluded.

## Gut–kidney axis

**Fig. 3:**
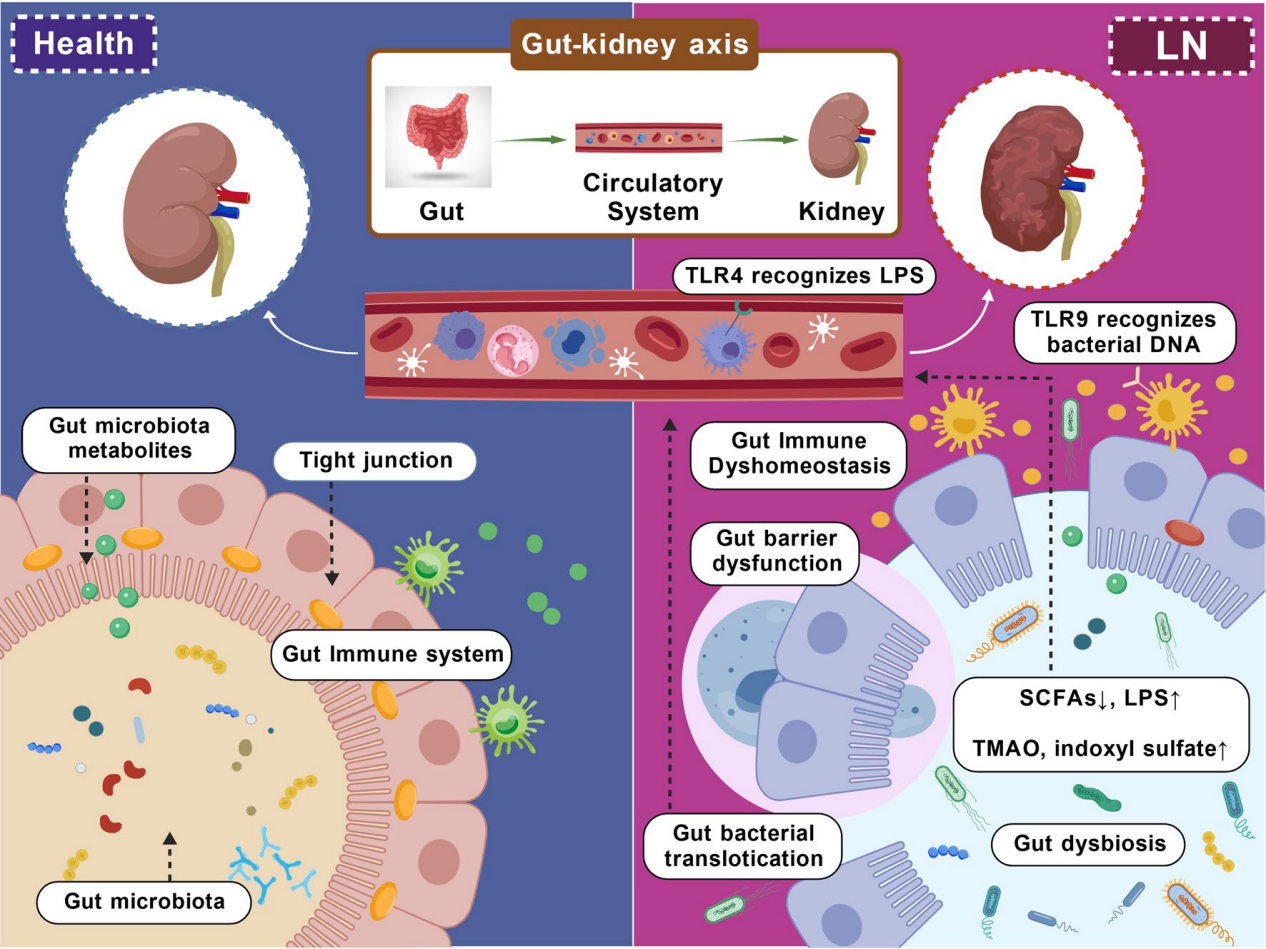
Role of the gut–kidney axis in LN pathogenesis. In LN, gut dysbiosis and compromised intestinal barrier integrity promote the translocation of microbial products (e.g., LPS) and metabolites (e.g., TMAO) into circulation. These substances drive systemic immune dysregulation by activating pattern recognition receptors and downstream signaling pathways. Additionally, microbiota-derived antigens directly activate autoreactive T cells and B cells via molecular mimicry, promoting pathogenic autoantibody production and immune complex formation. These complexes deposit in the kidneys, triggering local inflammation and progressive glomerular and tubular injury. This figure was created with BioGDP [13]. *SCFAs* short-chain fatty acids, *LPS* Lipopolysaccharide, *TMAO* trimethylamine N-oxide

The gut–kidney axis theory posits that the gut influences renal function and disease progression by regulating host metabolic and immune homeostasis (Fig. 3) [14].

### Structure and function of the gut–kidney axis

The gut–kidney axis links the gut microbiota ecosystem with renal physiological functions [15]. The components of the gut–kidney axis include the gut microbiota and its metabolites, the intestinal barrier, the gut immune system, the circulatory system, and the kidneys.

The gut microbiota comprises bacteria, fungi, viruses, and archaea, with bacteria predominantly from the *Firmicutes* and *Bacteroidetes* phyla [16, 17]. The intestinal barrier consists of an epithelial cell layer, tight junction proteins, and a mucus layer that maintains selective permeability [18–21]. Within this structure, the mucus layer contains secretory immunoglobulin A (sIgA), which is produced by plasma cells in gut-associated lymphoid tissue [22]. SIgA coats microorganisms, preventing their adhesion and epithelial penetration, thus serving as the first line of mucosal defense. In autoimmune diseases such as LN, abnormal sIgA secretion or function may contribute to intestinal barrier dysfunction and microbial translocation.

Gut-associated lymphoid tissue is a key component of mucosal immunity, recognizing and responding to gut-derived antigens [17, 23, 24]. The circulatory system transports microbial metabolites, including short-chain fatty acids (SCFAs), LPS, and trimethylamine N-oxide (TMAO), to the kidneys, and it transports renal metabolic byproducts and signaling molecules back to the gut, enabling bidirectional communication.

The gut–kidney axis performs two key functions, namely metabolic regulation and immune modulation. Gut microbes breakdown dietary fiber to produce beneficial SCFAs, which help regulate systemic energy and metabolic balance. Conversely, gut microbes may generate harmful compounds, such as TMAO, which can impair kidney function upon entering the bloodstream [8, 25]. Gut microbes also help maintain immune homeostasis and preserve intestinal barrier integrity, thereby preventing bacterial and toxin translocation.

When the intestinal barrier is compromised, bacterial and toxin translocation triggers systemic inflammation and exacerbates renal injury. In turn, inflammatory mediators released by damaged kidneys further disrupt the gut microbiota composition and barrier function, creating a vicious cycle.

### Dysregulation of the gut–kidney axis in LN

Accumulating evidence suggests that gut–kidney axis dysregulation is a key upstream mechanism in LN pathogenesis. Disruption of the intestinal barrier and microbiota dysbiosis facilitate the translocation of microorganisms or their byproducts, which activates innate immune pathways, such as TLR4, and molecular mimicry mechanisms, thereby driving systemic autoimmune responses [20]. Abnormal enrichment of specific microbiota, such as rumen bacteria, is closely correlated with LN disease activity [26]. Thus, targeting the gut–kidney axis represents a promising therapeutic strategy for LN [14].

#### Gut dysbiosis

Gut dysbiosis is a hallmark of LN [1, 9, 10]. LN patients and lupus mouse models (e.g., MRL/lpr mice) exhibit significant imbalances in the gut microbiota imbalances, which are typically characterized by a decreased *Firmicutes*/*Bacteroidetes* ratio compared with that of healthy controls [5, 27].

At the genus level, beneficial symbionts with immunoregulatory functions, such as *Lactobacillus*, are significantly reduced, diminishing their protective role in maintaining immune homeostasis and suppressing excessive inflammation [9, 28]. Similarly, the abundance of butyrate-producing and other anti-inflammatory SCFA-generating bacterial groups, such as *Faecalibacterium prausnitzii* and *Roseburia* spp., is also significantly reduced [9, 29]. Conversely, the proportion of *Bacteroidetes*, which possesses proinflammatory potential, abnormally increases [9, 30]. The abundance of *Proteobacteria*, which includes gram-negative pathogens, such as *Enterobacteriaceae*, is also increased. These alterations create conditions conducive to sustained systemic immune activation and inflammatory responses [31].

Overall, the gut microbiota undergoes marked structural alterations, including reduced beneficial bacteria, increased potentially harmful bacteria, and diminished α-diversity, reflecting severely compromised intestinal microecological stability. This imbalance not only results from LN but also exacerbates renal immune inflammation and disease progression [32].

Transplanting the gut microbiota from SLE-prone mice into germ-free mice induces SLE-like immune responses in recipients, including elevated anti-dsDNA antibody levels, expansion of B cells and plasma cells, an imbalanced Treg/Th17 ratio, and type I interferon pathway activation. Mechanistically, the microbiota of SLE-prone mice upregulates the expression of several susceptibility genes, such as IRF7 and CSK. These findings suggest that gut dysbiosis may act as an independent environmental factor that directly drives SLE immune responses and disease progression, providing critical evidence for microbiota-targeted therapies [33].

Animal studies have confirmed that supplementation with probiotics, such as *Lactobacillus*, or modulation of the microbial composition helps mitigate renal inflammation [28]. These microbial alterations are closely linked to impaired intestinal barrier function, heightened systemic inflammation, and abnormal autoimmune activation, collectively driving LN onset and progression [34]. Additionally, dysregulated microbial metabolic functions, such as enhanced carbohydrate degradation and oxidative phosphorylation, promote inflammatory responses and autoantigen formation [35].

#### Intestinal barrier injury and metabolic endotoxemia

Dysbiosis and associated metabolic changes compromise intestinal barrier integrity [36]. In LN patients and mouse models, the expression of colonic epithelial tight junction proteins, such as ZO-1 and claudin-1, is significantly downregulated, leading to increased intestinal permeability [26, 26, 32]. Clinical studies have confirmed elevated levels of fecal calprotectin, a biomarker of intestinal barrier dysfunction indicating active mucosal inflammation, in LN patients [37]. These alterations reflect pronounced intestinal leakage.

Impaired intestinal barrier function facilitates the translocation of some bacterial products such as LPS into circulation, leading to metabolic endotoxemia. Circulating pathogen-associated molecular patterns (PAMPs), including LPS, activate immune cells, such as monocytes, macrophages, and dendritic cells, as well as renal resident cells, including mesangial cells and podocytes, via pattern recognition receptors (PRRs), primarily TLR4. TLR4 activation induces the release of proinflammatory cytokines, including type I interferon, IL-6, and TNF-α, triggering systemic inflammation. Notably, podocytes themselves express TLR4, and direct LPS stimulation exacerbates podocyte injury and proteinuria [38, 39].

#### Dysregulation of microbial metabolites

Gut microbiota-derived metabolites play crucial regulatory roles in immune function [16, 38]. In LN, disturbed microbial metabolic activity leads to reduced levels of protective metabolites. SCFA deficiency, particularly in the presence of butyrate, impairs intestinal barrier integrity and immune homeostasis. Butyrate activates G protein-coupled receptors such as GPR43 and GPR109A, and inhibits the expression of histone deacetylases (HDACs), which promotes immunomodulatory Treg differentiation, suppresses proinflammatory Th17 responses, and enhances intestinal barrier function. However, these beneficial effects are limited by decreased butyrate production in LN [30, 40].

Concurrently, harmful metabolites accumulate [26, 41, 42]. With declining renal function, gut microbiota-derived metabolites, such as p-cresyl sulfate (derived from tyrosine and phenylalanine breakdown) and indoxyl sulfate (derived from tryptophan breakdown), accumulate more readily [43–45]. These substances induce oxidative stress in renal tubular epithelial cells, promote inflammatory cytokine secretion, trigger apoptosis, and exacerbate renal inflammation via NLRP3 inflammasome activation.

Additionally, TMAO produced by the gut microbial metabolism of choline and L-carnitine, is significantly elevated in LN patients. TMAO promotes macrophage polarization toward the proinflammatory M1 phenotype, intensifies local renal inflammation, and may directly induce podocyte injury and glomerulosclerosis.

#### Molecular mimicry and autoimmune activation

Molecular mimicry reveals how the gut microbiota disrupts systemic immune tolerance. Certain gut symbionts express antigenic epitopes structurally similar to self-antigens, causing immune responses directed against foreign antigens to mistakenly attack host tissues. This phenomenon has been experimentally validated for numerous bacterial strains.

For example, the LPS domain of *Ruminococcus gnavus* cross-reacts with dsDNA, a characteristic lupus autoantigen, directly activating anti-dsDNA antibody-producing B cells and promoting their differentiation into plasma cells [26, 37]. Specific surface antigens of *Enterococcus gallinarum* resemble β_2_-glycoprotein I (β_2_GPI), a target antigen in antiphospholipid syndrome, inducing pathogenic antiphospholipid antibody production. Protein sequences expressed by certain *Bacteroides* strains share homology with the Ro60 antigen associated with Sjögren's syndrome and lupus, potentially disrupting autoimmune tolerance to this antigen [46–48].

This cross-reactivity directly activates autoreactive B cells and activates autoreactive T cells through the presentation of mimetic epitopes, providing essential costimulatory signals to B cells and establishing a sustained autoimmune response cycle [30]. The resulting autoantibodies, such as anti-dsDNA antibodies, form circulating immune complexes that are deposited in the glomerular basement membrane, activate the complement system and recruit inflammatory cells, ultimately triggering renal inflammation and tissue injury [32, 49].

Animal studies have further validated this mechanism. Lupus-prone mouse models have been colonized with specific proinflammatory bacterial strains, such as *Veillonella* species, resulting in markedly intensified autoimmune responses and renal pathology, confirming that the gut microbiota directly contributes to LN pathogenesis through molecular mimicry.

## Modern interpretation of the TCM theory of the spleen–kidney relationship from the perspective of the gut–kidney axis

**Fig. 4:**
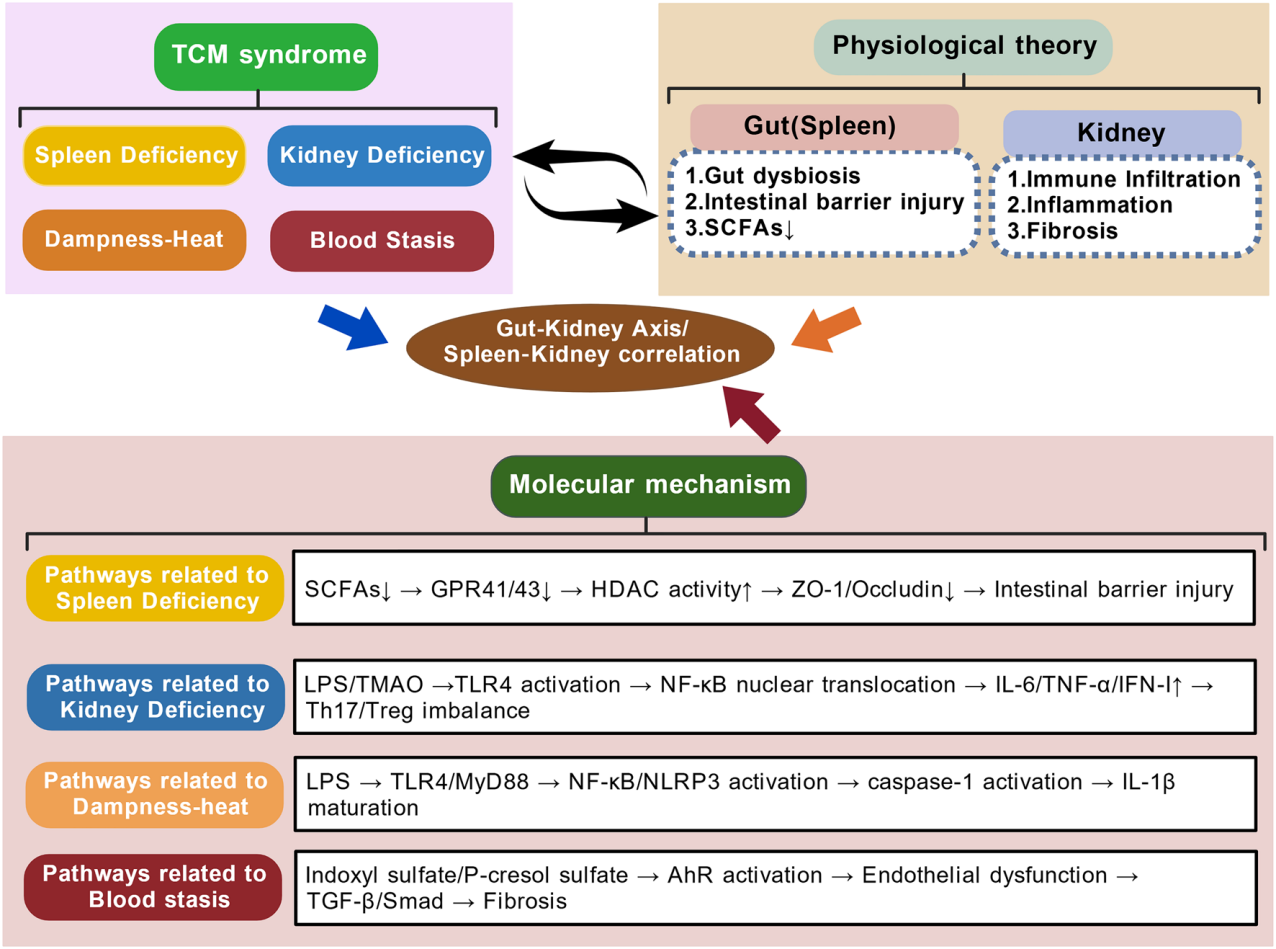
TCM syndromes and gut–kidney axis molecular pathways in LN. This figure links four core TCM syndromes—spleen deficiency, kidney deficiency, dampness-heat, and blood stasis—to their associated molecular pathways. Spleen deficiency correlates with SCFA/GPR/HDAC signaling [50–52]. Kidney deficiency correlates with TLR4/NF-κB and TGF-β/Smad pathways [53, 54]. Dampness-heat correlates with NLRP3 inflammasome activation [55–57]. Blood stasis correlates with Wnt/β-catenin and AhR pathways [58–61]. This figure was created with BioGDP [13]. *HDACs* histone deacetylases, *AhR* aryl hydrocarbon receptor

This chapter interprets the core concepts of TCM pathogenesis in LN, including the spleen, kidney, dampness-heat, and blood stasis, within the framework of the gut–kidney axis. This chapter also aims to establish correlations between TCM theory and modern biological mechanisms. This approach provides a verifiable biological basis for TCM pathogenesis and lays a theoretical foundation for elucidating the multitarget, holistic regulatory pharmacological mechanisms of TCM in LN treatment (Fig. 4).

### TCM pathogenesis of LN

In TCM, the complex clinical manifestations of LN are often categorized into various syndromes, such as Yin-Yang toxin, water swelling, and consumptive disease [62]. Yin-Yang toxin refers to acute febrile conditions characterized by pharyngeal pain and cutaneous rashes, which are associated with the core pathogenesis of toxin stagnation and blood stasis in the vessels. This concept closely corresponds to the systemic inflammatory state and immune hyperactivation observed during acute flares of SLE, including the elevation of proinflammatory cytokines and immune complex deposition in target organs, such as the glomerulus. The core pathogenesis of LN lies in dysfunction of the spleen and kidney, which is frequently complicated by dampness-heat and blood stasis [62].

The Spleen, regarded as the root of acquired essence, governs the transformation and transportation of nutrients and fluids, and it is responsible for ascending clear substances. Spleen deficiency can lead to internal retention of dampness and fluid, as well as downward leakage of nutrients. The kidney, as the root of innate essence, governs water metabolism and stores essence. Kidney deficiency disrupts fluid metabolism and generates internal heat, which manifests as edema, proteinuria, and chronic inflammation [63, 64].

Physiologically, the spleen and kidney mutually support each other. Pathologically, the spleen and kidney form a vicious cycle, in which a spleen disorder affects the kidney and a kidney disorder affects the spleen. This interdependent relationship may represent a key intrinsic mechanism underlying the chronic and difficult-to-treat nature of LN [65].

### Modern interpretation of TCM theory based on the gut–kidney axis

The gut–kidney axis theory provides modern scientific support for related TCM concepts, as it translates the holistic understanding of diseases into observable and verifiable modern pathological mechanisms.

#### Relationship between the TCM spleen and intestinal homeostasis in TCM

The ability of the spleen to clear substances in TCM, is closely linked to intestinal digestion and absorption, microbial ecology, and the integrity of the intestinal epithelial barrier. Consequently, spleen deficiency may correspond to gut dysbiosis, reduced SCFA production, and intestinal barrier injury, leading to impaired nutrient absorption [65].

From a molecular mechanism perspective, spleen deficiency manifesting as gut dysbiosis may reduce the production of butyrate and other SCFAs, thereby weakening their ability to activate G protein-coupled receptors, such as GPR41 and GPR43, on intestinal epithelial cells and inhibit HDACs [50–52]. This signaling disruption directly leads to the downregulation of the expression of tight junction proteins, including ZO-1 and occludin, and increased intestinal permeability. These changes validate the TCM concept that the spleen fails to transport and ascend clear essences [50, 52].

Clinical and experimental studies have indicated that astragalus polysaccharides, the primary active component of the spleen-tonifying herb *Astragalus membranaceus*, significantly increase the abundance of beneficial gut bacteria, upregulate the expression of tight junction proteins (such as ZO-1), and reduce circulating LPS levels [66, 67]. These findings confirm the theoretical principle that the spleen governs the root of acquired essence.

#### Association between the TCM kidney and the renal immune microenvironment in TCM

The TCM concept that the kidney governs water is directly linked to renal filtration and excretory functions. In the context of immune inflammation in LN, kidney deficiency can be further interpreted as an imbalance in local renal immune homeostasis, manifested as local kidney inflammation, abnormal immune cell infiltration, and the activation of profibrotic pathways [62, 68].

Mechanistically, kidney deficiency involves persistent activation of pattern recognition receptors, such as TLR4, on renal resident cells by gut-derived microbial products, including LPS and TMAO, leading to downstream NF-κB signaling activation and increased production of proinflammatory cytokines, such as IL-6, TNF-α, and type I interferons. This creates a proinflammatory microenvironment that promotes Th17 cell differentiation while suppressing Treg cell function, an immunological imbalance that corresponds to the TCM concept of kidney Yin deficiency, which generates internal heat. Concurrently, activated TGF-β and Smad signaling drive renal fibrosis, reflecting the pathological progression from kidney deficiency to tissue damage [16, 53, 54].

Modern pharmacological research provides supporting evidence for this concept. Catalpol, a key active component of the kidney-tonifying herb *Rehmannia glutinosa*, has been reported to regulate the balance of local renal T-cell subsets, inhibit inflammatory pathways (such as NF-κB), and alleviate glomerulosclerosis. These findings verify the role of tonifying the kidney in modulating the renal immune microenvironment [69].

#### Dampness-heat and systemic inflammation

The clinical manifestations of TCM dampness-heat syndrome closely resemble the chronic systemic inflammatory state driven by gut–kidney axis dysregulation [70]. The modern material basis of dampness-heat syndrome may correspond to increased intestinal barrier permeability resulting from spleen deficiency, which allows the translocation of microbial products (such as LPS and harmful metabolites, including TMAO) into the bloodstream [55].

At the molecular level, these dampness-heat toxins act as PAMPs that bind to TLR4 on immune cells, triggering MyD88-dependent signaling cascades that activate the NF-κB and NLRP3 inflammasome pathways. NF-κB nuclear translocation promotes the transcription of proinflammatory cytokines, including IL-1β, IL-6, and TNF-α, while NLRP3 inflammasome activation leads to caspase-1 cleavage and IL-1β maturation. These molecular events manifest clinically as elevated inflammatory markers and correspond to the heat signs in TCM [56, 57].

The persistent nature of this inflammatory state, driven by continuous microbial product translocation, mirrors the TCM concept of dampness as a lingering, difficult-to-resolve pathogenic factor. Clinically, this state manifests as elevated proinflammatory cytokines, which correspond to the heat signs in TCM [55, 65, 71].

The effects of heat-clearing and dampness-removing herbs directly involve these mechanisms. Berberine, the primary alkaloid of *Coptis chinensis*, has been widely demonstrated to exert anti-endotoxic activity, inhibit TLR4 and NF-κB signaling, and reduce serum levels of IL-6 and TNF-α. These findings provide molecular pharmacological evidence for the TCM therapeutic principle of clearing heat and drying dampness [72].

#### Blood stasis, and renal microcirculation and fibrosis

In the course of LN, blood stasis is both a product of the aforementioned pathological processes and a factor that exacerbates renal injury. The modern medical implications of blood stasis include local renal microcirculatory disorders, hypercoagulable states, endothelial injury, and progressive fibrosis [73–75].

From a mechanistic perspective, the material basis of blood stasis can be attributed to gut-derived uremic toxins (including indoxyl sulfate and p-cresol sulfate) and chronic inflammatory mediators that induce endothelial dysfunction through multiple pathways [55, 74]. Indoxyl sulfate activates aryl hydrocarbon receptors in endothelial cells, increasing oxidative stress and reducing nitric oxide bioavailability, leading to impaired vasodilation and a procoagulant state [58]. Moreover, sustained inflammatory signaling activates the TGF-β and Smad pathways and the Wnt and β-catenin pathways in renal fibroblasts and tubular epithelial cells, promoting extracellular matrix deposition and driving glomerulosclerosis and interstitial fibrosis [59–61]. These molecular events, including endothelial activation, microthrombosis, and progressive matrix accumulation, represent the modern pathological correlates of blood stasis obstructing collaterals in TCM [61, 73, 74, 76].

On the basis of the gut–kidney axis theory, the material basis of blood stasis can be partially attributed to gut-derived uremic toxins such as indoxyl sulfate and p-cresol sulfate, as well as chronic inflammatory mediators. These substances induce endothelial dysfunction, promote a procoagulant state and imbalance in the fibrinolytic system, and persistently activate profibrotic pathways (such as the TGF-β and Smad pathways), ultimately leading to glomerulosclerosis and interstitial fibrosis [76].

Salvianolic acid B, the primary active component of the blood-activating and stasis-resolving herb *Salvia miltiorrhiza*, has been demonstrated to improve renal microcirculation, inhibit platelet aggregation, protect endothelial cells, and suppress renal interstitial fibrosis. These effects systematically elucidate the modern therapeutic objectives of activating blood and dredging collaterals [77].

### TCM spleen–kidney correlation theory and the gut–kidney axis

The TCM theory of the spleen–kidney correlation aligns closely with the modern medical concept of the gut–kidney axis in terms of pathological mechanisms, revealing a bidirectional relationship in LN disease progression [65]. Spleen disorder affecting the kidney manifests as spleen deficiency, which involves gut dysbiosis and intestinal barrier injury. This leads to the systemic dissemination of endotoxins and inflammatory mediators, directly or indirectly exacerbating kidney deficiency characterized by renal immune inflammation and injury. Conversely, kidney disorders affecting the spleen reflect how kidney deficiency, as observed in renal failure or uremia, further aggravates gut dysbiosis and compromises intestinal barrier function, thereby intensifying spleen deficiency [65].

The interpretation of the spleen–kidney correlation theory according to the gut–kidney axis provides a modern theoretical foundation for the TCM approach of fortifying the spleen and tonifying the kidney to treat LN [65]. LN pathogenesis is characterized by spleen–kidney deficiency throughout the disease course, presenting as a syndrome of asthenia in origin and sthenia in superficiality with intermingled deficiency and excess.

The method of strengthening the spleen and nourishing the kidney, grounded in the TCM theory of consolidating the root and cultivating the primary, is embodied in the Jianpi–Zishen formula. This formula, modified from Liuwei Dihuang Decoction, consists of the following eight herbs: *Astragalus membranaceus*, processed *Rehmannia glutinosa*, *Dioscorea opposita*, *Atractylodes macrocephala*, *Rubus chingii*, *Cuscuta chinensis*, *Rosa laevigata*, and *Poria cocos*. Previous clinical studies have demonstrated that this formula reduces proteinuria, improves renal function, and regulates immune factor levels in patients with SLE [78].

Modern pharmacological studies have provided experimental evidence supporting the use of the spleen–kidney tonifying approach. *Astragalus membranaceus* increases the abundance of probiotics (such as *Lactobacillus* and *Bifidobacterium)*, reduces inflammatory cytokine levels, and repairs intestinal barrier damage. Processed *Rehmannia glutinosa* promotes the proliferation of *Bifidobacterium* and *Lactobacillus*, and it reduces oligosaccharide content, which facilitates SCFA release. Dioscorea opposita regulates the gut microbiota composition, inhibits proinflammatory cytokine secretion, and has anticolitic effects. Atractylodes macrocephala enhances probiotic adhesion to the intestinal mucosa, inhibits pathogenic bacterial proliferation, and repairs the intestinal immune barrier. Poria cocos increases the relative abundance of *Bacteroidetes* and *Alistipes*, reduces the abundance of *Firmicutes*, and ameliorates renal pathological damage [78].

## Multimechanistic modulation of the gut–kidney axis by TCM

**Fig. 5:**
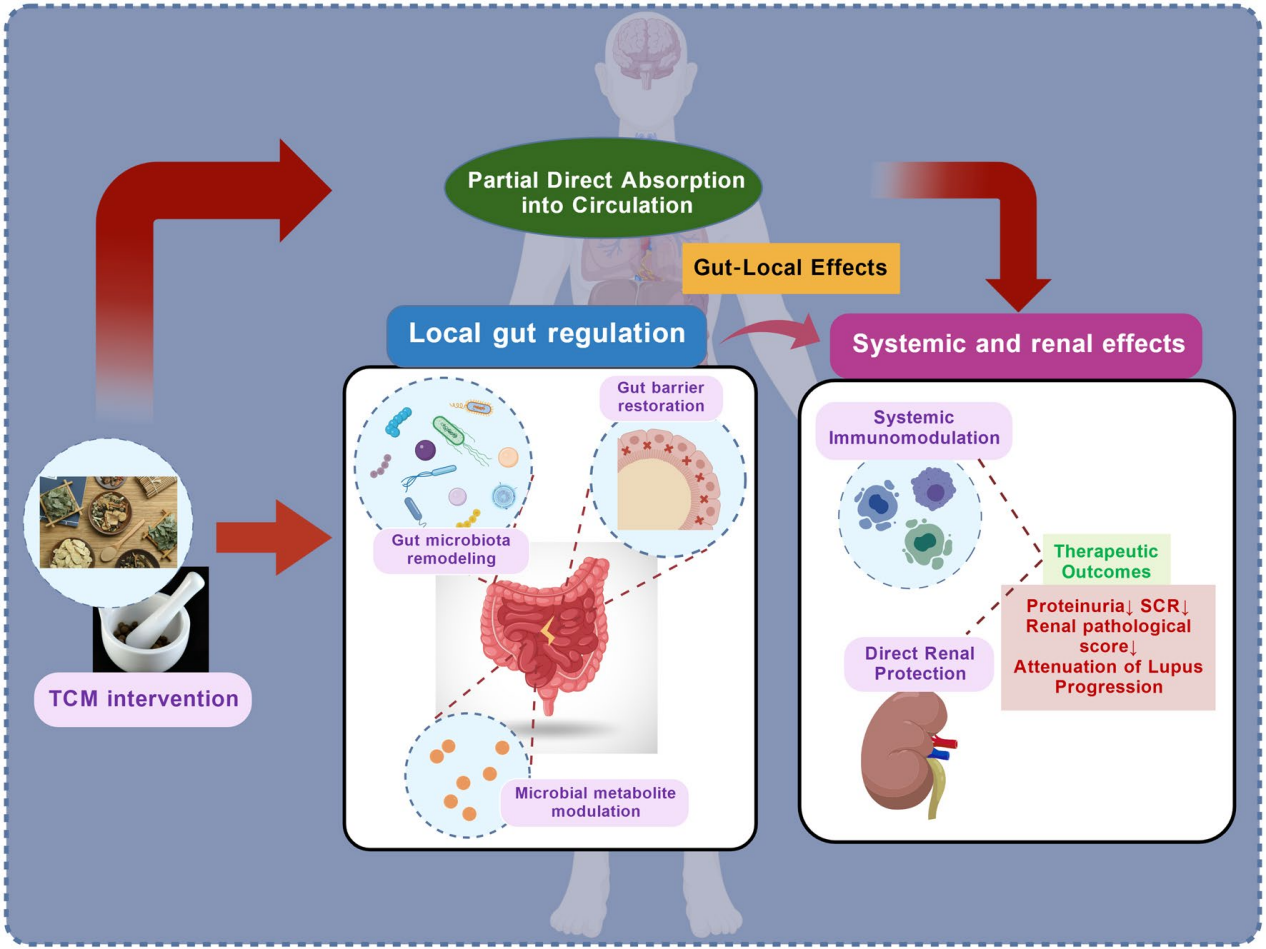
Multimechanism regulation of the gut–kidney axis by TCM. After oral administration, TCM active components act in the intestine through four synergistic mechanisms. (i) Reshaping gut microbiota composition: increasing microbial diversity and promoting SCFA-producing bacteria. (ii) Enhancing intestinal barrier function: upregulating ZO-1 and occludin, reducing intestinal permeability, and limiting pathogen translocation. (iii) Regulating microbial metabolites: promoting SCFAs while inhibiting LPS and TMAO. These metabolites enter the circulation and exert systemic effects. Together with direct renal actions of TCM, these mechanisms (iv) suppress systemic immune-inflammatory responses, reducing renal immune cell infiltration, alleviating local inflammation, and mitigating histopathological injury. This figure was created with BioGDP [13]

On the basis of the theories discussed above, TCM treatment for LN is a multimechanism systemic intervention strategy targeting the gut–kidney axis. Although LN has unique autoimmune characteristics, its core pathological mechanisms, including gut dysbiosis, intestinal barrier injury, the dysregulation of microbial metabolites, and the resulting systemic immune inflammation, are similar to those of renal disease models, such as chronic kidney disease (CKD). Extensive research has systematically elucidated the mechanisms by which TCM improves abnormalities associated with CKD (Fig. 5).

This chapter summarizes the main approaches by which TCM intervenes in the gut–kidney axis in the context of CKD, thereby providing mechanistic evidence to clarify how TCM treats LN through holistic regulation.

### Reshaping the gut microbiota composition

Gut dysbiosis affects the function of the gut–kidney axis. TCM treatment strategies often include modulation of the gut microbiota, specifically by promoting beneficial bacterial proliferation and inhibiting harmful bacterial growth to improve the gut microenvironment.

Studies have shown that many TCM formulas and their active components, such as Yi-Shen-Hua-Shi (YSHS) granules, Bupi Yishen formula (BPYSF), and various polysaccharides and flavonoids, effectively increase the abundance of beneficial gut bacteria, including *Akkermansia*, *Faecalibacterium*, and *Lactobacillus* [76, 79, 80]. These microbial populations contribute to reducing inflammation and repairing the intestinal mucosa. Moreover, these TCM interventions inhibit the overgrowth of opportunistic pathogens such as *Escherichia-Shigella* and *Clostridium innocuum* [61].

Additionally, certain TCM herbs, such as Panax notoginseng saponins and Huangkui capsules (HKC), promote the proliferation of butyrate-producing bacteria including Clostridium sensu stricto 1 and Bifidobacterium, thereby increasing the production of beneficial substances such as SCFAs [76, 81]. Research has also indicated that some TCM formulas, such as Jian-Pi-Yi-Shen formula, modulate the structure and function of the gut microbiota, restoring microbial cooperation associated with beneficial metabolic processes (such as flavonoid synthesis) and systematically reducing uremic toxin production [82]. Therefore, modulating the gut microbiota composition is crucial for the therapeutic effects of TCM and the subsequent amelioration of gut and renal disorders.

### Enhancing intestinal barrier function

TCM regulates the intestinal microbiota and enhances intestinal barrier function through multiple mechanisms, which is crucial for preventing intestinal toxins from entering the bloodstream and reducing renal injury. The protective effects of TCM primarily involve strengthening the intestinal physical barrier and modulating the local gut immune environment.

On the one hand, various active components in TCM (including curcumin, salvianolic acid B, and Astragalus polysaccharides) and formulas (such as YSHS granules and the combination of *Astragalus membranaceus* and *Salvia miltiorrhiza* ([AS]), directly promote the production of intestinal intercellular junction proteins [76, 79, 83, 84]. This helps repair and reinforce the intestinal mucosal structure, thereby reducing intestinal permeability.

On the other hand, TCM indirectly strengthens barrier function by modulating intestinal immune responses. For example, TCM formulas (such as BPYSF) activate type 3 innate lymphoid cells and their downstream IL-22 signaling pathway, promoting the regeneration and repair of intestinal epithelial cells [80]. TCM also promotes the production of beneficial metabolites, including spermidine and SCFAs, by the gut microbiota. These substances mediate the protective effects of TCM on the intestinal barrier [85, 86].

Certain TCM components—such as the graminan-type fructan ABPW1 and Moutan Cortex polysaccharides—promote the production of SCFAs, such as propionic acid and isobutyric acid, by the gut microbiota [87, 88]. These SCFAs induce macrophage polarization from the proinflammatory M1 phenotype to the anti-inflammatory M2 phenotype, thereby modulating the immune microenvironment of the intestinal barrier.

The aforementioned outcomes effectively reduce the entry of LPS and uremic toxins, such as indoxyl sulfate and p-cresyl sulfate, from the intestine into the systemic circulation, thereby mitigating the persistent renal damage caused by these gut-derived toxins [85, 89–91].

### Modulating microbial metabolites

TCM modulates the composition and function of gut microbiota metabolites. As key signaling molecules in the gut–kidney axis, dysregulation of these metabolites directly contributes to and exacerbates renal injury progression. TCM acts through multiple mechanisms, promoting beneficial metabolite production while reducing harmful product accumulation, thereby achieving renal protection.

TCM enhances the generation and function of protective metabolites. Studies have shown that polysaccharide components, including Astragalus polysaccharides, Achyranthes bidentata polysaccharides, and fucoidan, significantly promote the production of SCFAs, such as propionate and butyrate, by the gut microbiota [87, 92–94]. These SCFAs inhibit downstream renal inflammatory signaling pathways by activating G-protein coupled receptors including GPR43 and GPR109A [30, 40]. Moreover, microbiota-derived spermidine activates cellular autophagy, and retinoic acid modulates IL-22 secretion, collectively delaying CKD progression.

TCM effectively inhibits the accumulation and toxicity of harmful metabolites. For example, HKC reduces intestinal accumulation of indole-type toxins by modulating the tryptophan metabolic pathway [81]. Zuogui-Jiangtang-Yishen decoction and Yiqi-Huoxue-Jiangzhuo formula inhibit TMAO production [95, 96]. Additionally, Suyin detoxification granules decrease the abundance of TMAO-related bacterial genera and reduce circulating TMAO levels, thereby suppressing TMAO-induced renal tubular ferroptosis [97].

TCM synergistically regulates multiple key metabolic pathways. For example, the AS and YSHS granules herb pair interferes with critical pathways, including sphingolipid metabolism, glycerophospholipid metabolism, and arachidonic acid metabolism, modulating the production and balance of bioactive substances, such as ceramide and sphingosine [79, 84]. Ootheca mantidis exerts its effects through the regulation of glutamine metabolism, whereas Fufang-Zhenzhu-Tiaozhi formula and Moutan Cortex polysaccharides alleviate renal inflammation and fibrosis by promoting intestinal SCFA production [88, 98, 99]. Moreover, magnesium lithospermate B interferes with bile acid metabolism to modify bile acid composition, enhancing gut–kidney signaling efficiency [100]. Through the modulation of these key metabolic pathways, TCM helps to systematically control renal inflammation, slow fibrosis progression, and achieve holistic improvement.

### Suppressing local and systemic immune inflammation

Persistent inflammation is a core driver of kidney injury progression. TCM modulates multiple targets within immune–inflammatory signaling pathways, effectively alleviating both local renal and systemic inflammatory responses and slowing CKD progression.

Multiple TCM active components and formulas inhibit key inflammatory signaling pathways. Baicalin targets critical signaling molecules (including NF-κB, TGF-β and Smad) and the NLRP3 inflammasome, reducing the levels of proinflammatory cytokines, such as TNF-α and IL-6 [101]. Tangshen formula suppresses the activity of JNK and NF-κB signaling, decreasing the release of chemokines, including MCP-1 [102]. Xiaoyu Xiezhuo decoction indirectly inhibits NF-κB activation by modulating aldosterone, the mineralocorticoid receptor (MR), and the serum- and glucocorticoid-inducible kinase-1 (SGK-1) axis [103]. Furthermore, spermidine in modified Huangfeng decoction regulates the PI3K, AKT, and mTOR pathways to activate podocyte autophagy, and alleviate inflammation and fibrotic injury [104, 105]. TCM also regulates immune cell subsets, with various formulas correcting immune imbalance by modulating the Th17 and Treg cell balance.

TCM affects the gut–kidney axis by modulating the gut microbiota, restoring intestinal barrier function, improving metabolic homeostasis, and suppressing inflammatory responses [106]. These mechanisms, which have been validated in CKD treatment, are also applicable to gut–kidney axis dysregulation in LN. This provides a modern scientific basis for the TCM theory of spleen–kidney correlation and supports the feasibility of treating LN through microbiota regulation. The following sections focus specifically on LN. This chapter aims to systematically elucidate the scientific rationale underlying the holistic, multimechanism intervention of TCM in regulating the gut–kidney axis.

### Synergistic mechanisms of the gut–kidney axis regulation

TCM regulation of the gut–kidney axis is not a combination of the four mechanisms discussed above but rather a dynamic process involving intrinsic causal sequences and mutual feedback loops. This process typically begins with TCM remodeling of the gut microbiota composition. For example, polysaccharide components in TCM serve as prebiotics, promoting the proliferation of beneficial bacteria, such as *Lactobacillus* and butyrate-producing bacteria.

This microbial remodeling directly leads to alterations in microbial metabolites, with a significant increase in the production of SCFAs, particularly butyrate. On the one hand, increased SCFAs provide energy for intestinal epithelial cells and activate receptors (such as GPR43), upregulating tight junction protein expression and thereby repairing and enhancing intestinal barrier function. On the other hand, upon entering circulation, SCFAs regulate the differentiation of peripheral immune cells, promoting Treg cell generation while inhibiting Th17 cell responses, thereby systemically suppressing immune inflammation.

As the intestinal barrier is repaired, the translocation of proinflammatory products (such as LPS) is reduced, leading to decreased systemic inflammation levels. This alleviates the destructive effects of inflammatory factors on the gut microbiota and barrier, resulting in the formation of a positive, self-reinforcing and beneficial cycle. Therefore, the therapeutic efficacy of TCM stems from its ability to initiate and maintain this multilayered synergistic repair network.

## TCM interventions targeting the gut–kidney axis in LN

**Fig. 6:**
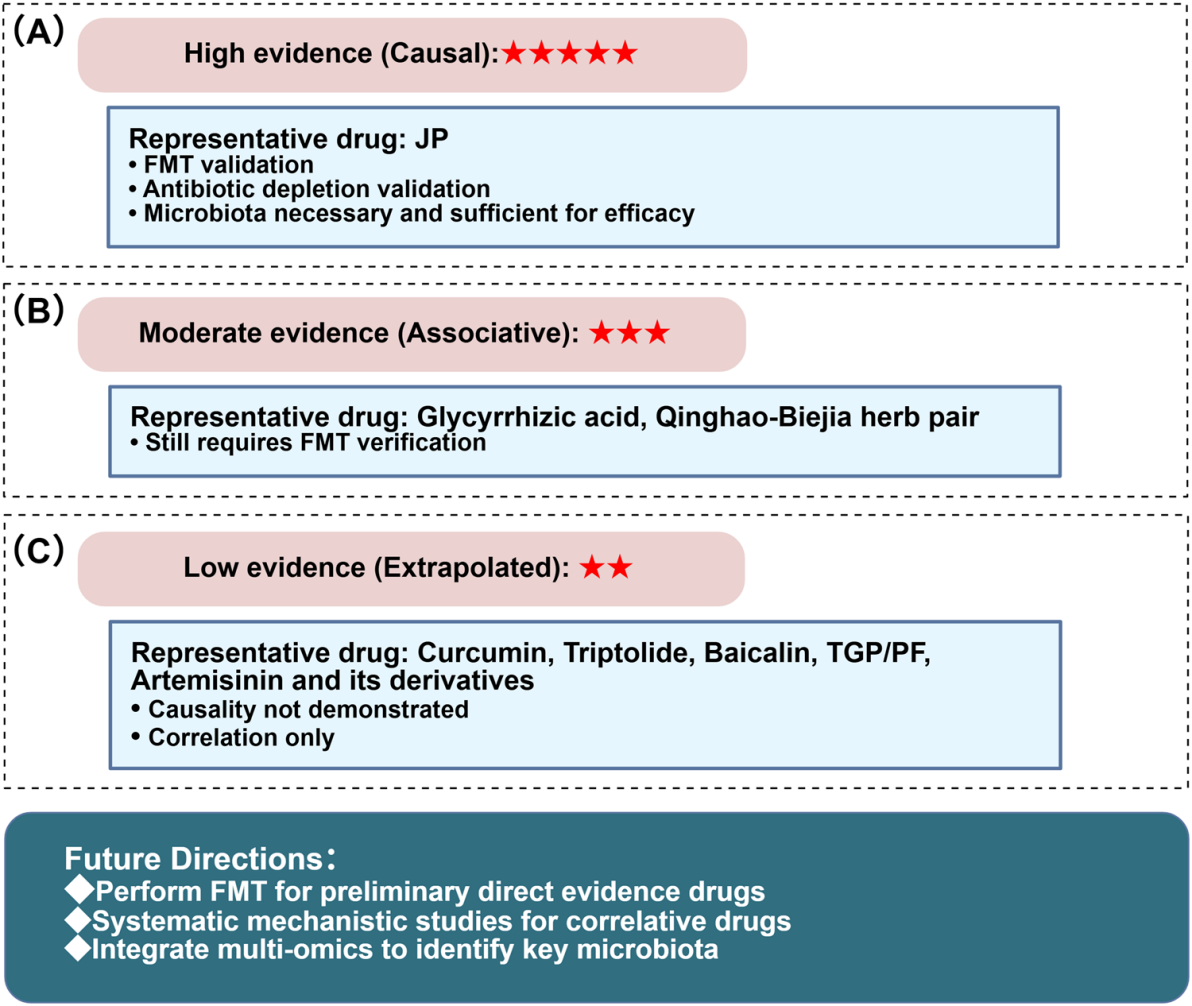
Evidence levels for TCM interventions targeting the gut–kidney axis in LN. **A** High evidence (causal): JP is the only intervention with complete causal validation. FMT or antibiotic depletion experiments have demonstrated that the gut microbiota is both necessary and sufficient for its efficacy. **B** Moderate evidence (associative): Glycyrrhizic acid and the Qinghao-Biejia herb pair exhibit renoprotective effects associated with gut microbiota modulation in LN models, but direct causal validation is lacking. **C** Low evidence (extrapolated): Several TCM monomers show efficacy in LN models and established microbiota-regulating effects in other diseases. However, in LN models, only correlations between microbiota changes and therapeutic effects have been observed; causality has not been established. This figure was created with BioGDP [13]. *JP* Jieduquyuziyin prescription, *FMT* fecal microbiota transplantation, *TGP* total glucosides of paeony, *PF* paeoniflorin

While the previous chapter described the mechanisms by which TCM regulates the gut–kidney axis, this chapter systematically reviews TCM interventions for LN, categorizing the evidence into three levels, namely, high evidence (causal), moderate evidence (associative), and low evidence (extrapolated), as summarized in Fig. 6.

### High evidence (causal)

Studies on this class of TCM preparations have confirmed their therapeutic effects in LN. Fecal microbiota transplantation (FMT) experiments have established that gut microbiota modulation is both necessary and sufficient for their renoprotective efficacy (Table 1).

**Table 1:** High and moderate evidence TCM interventions targeting the gut–kidney axis in LN

TCM	Model	Administration	Key evidence	Evidence level	References
JP	MRL/lpr mice	● Oral gavage; 20 g/kg/day; 8–12 weeks	♦ Restores alpha diversity ♦ ↑ Bacteroides/Lactobacillus ♦ ↓ Prevotella ♦ ↓ Anti-dsDNA, IL-6, renal pathology	High	[107–109]
Glycyrrhizic Acid	MRL/lpr mice	● Intraperitoneal; 50 mg/kg/day; 8 weeks	♦ ↓ *Ruminococcus* abundance ♦ ↑ Intestinal SCFAs, ↓ circulating LPS ♦ Inhibits renal RTK-PKCα signaling	Moderate	[110]
Qinghao-Biejia herb pair	MRL/lpr mice	● Oral gavage; 10 g/kg/day; 12 weeks	♦ ↑ Alpha diversity, ↑ *Bacteroides/Parabacteroides/Odoribacter* ♦ ↓ *Clostridium/Ruminococcus* ♦ ↓ Proteinuria, Scr, TNF-α	Moderate	[111]

Evidence levels are defined as follows: High evidence (causal): the gut microbiota is both necessary and sufficient for therapeutic efficacy, as demonstrated by FMT or antibiotic depletion experiments. Moderate evidence (associative): renoprotective effects are associated with gut microbiota modulation in LN models, but causal validation via FMT or antibiotic depletion is lacking

*SCFAs* short-chain fatty acids, *RTK-PKCα* receptor tyrosine kinase-protein kinase C alpha, *Scr* serum creatinine, *JP* Jieduquyuziyin prescription, *FMT* fecal microbiota transplantation

Jieduquyuziyin prescription (JP) is among the most studied TCM formulas for treating lupus. JP is composed of *Rehmannia glutinosa* Radix, *Trionyx sinensis* Carapax, *Cimicifuga foetida* Rhizoma, *Hedyotis diffusa*, *Artemisia annua* Herba, *Centella asiatica* Herba, *Paeonia lactiflora* Radix Rubra, *Coix lacryma-jobi* Semen, *Citrus medica* var. *sarcodactylis* Fructus, and *Glycyrrhiza uralensis* Radix et Rhizoma. In MRL/lpr mice, JP restores alpha diversity and corrects the imbalance in the gut microbiota by increasing the abundance of beneficial bacteria, including *Bacteroides*, *Odoribacter*, *Ruminococcus*, and *Lactobacillu*s, while decreasing the abundance of pathogenic taxa, such as *Prevotella*, *Parabacteroides*, and *Rikenella* [107–109].

This microbial remodeling underlies the renoprotective effects of JP. FMT from JP-treated mice to recipient LN mice results in therapeutic benefits, including reduced anti-dsDNA antibodies, lower IL-6 levels, and improved renal injury. Conversely, antibiotic depletion of the gut microbiota or transplantation of the microbiota from untreated mice completely abolishes the efficacy of JP [107–109]. These FMT experiments confirm that the gut microbiota is both necessary and sufficient for the therapeutic effects of JP, establishing the highest level of causal evidence.

### Moderate evidence (associative)

This category includes TCM interventions with renoprotective effects linked to gut microbiota modulation in LN models. However, causal validation through FMT or antibiotic experiments is lacking (Table 1).

Glycyrrhizic acid, derived from Glycyrrhiza uralensis, protects against renal injury in MRL/lpr mice by regulating the gut–kidney axis. Glycyrrhizic acid reduces the abundance of *Ruminococcus*, increases SCFA levels, decreases circulating LPS levels, and inhibits renal RTK-PKCα signaling, alleviating immune complex deposition and fibrosis [110]. However, direct causal validation in LN models is still needed.

The efficacy of the Qinghao-Biejia herb pair is comparable to that of glucocorticoids in LN models. The Qinghao-Biejia herb pair increases alpha diversity and the abundance of beneficial bacteria, including *Bacteroides*, *Parabacteroides*, and *Odoribacter,* while reducing the abundance of pathogenic taxa, such as *Clostridium* and *Ruminococcus* [111]. These changes correlate with reduced proteinuria, serum creatinine, and TNF-α levels. The active constituents of the Qinghao-Biejia herb pair—artemisinin and dihydroartemisinin (DHA)—modulate the balance between Tregs and Th17 cells and inhibit NF-κB signaling, suggesting the combined role of the microbiota and immune regulation. Nevertheless, FMT experiments are needed to establish causality.

In summary, JP represents the only intervention with complete causal validation, while the effects of glycyrrhizic acid and Qinghao-Biejia need further studies to confirm the microbiota-mediated mechanisms involved.

### Low evidence (extrapolated)

TCM interventions in this category show clear efficacy in LN models and established gut microbiota-regulating effects in other diseases, but direct evidence linking these observations in LN is lacking (Tables 2, 3).

1. Curcumin

**Table 2:** Basic information of low evidence TCM interventions targeting the gut–kidney axis in LN

Herb source	Active ingredient	Model	Administration	References
*Curcuma longa* Rhizoma	Curcumin	MRL/lpr mice	● Dietary admixture; 500 mg/kg/day; 12 weeks	[115–121, 126–129]
*Tripterygium wilfordii* Radix	Triptolide	MRL/lpr mice	● Oral gavage; 0.1–0.2 mg/kg/day; 8–12 weeks	[134, 135, 137, 139, 149]
	Celastrol	B6/gld mice	● Intraperitoneal; 1 mg/kg/day; 8 weeks	[141, 143–148]
	Tripterygium Glycosides	LN patients (meta-analysis)	● Oral tablet; 60–120 mg/day; 6–12 months	[140, 151]
*Scutellaria baicalensis* Radix	Baicalin	MRL/lpr mice	● Intraperitoneal; 50–100 mg/kg/day; 8 weeks	[101, 153, 156–160]
	Baicalein	Pristane-induced lupus mice	● Intraperitoneal; 50 mg/kg/day; 4 weeks	[154, 155]
*Paeonia lactiflora* Radix Alba	TGP	MRL/lpr mice	● Oral gavage; 100–200 mg/kg/day; 8–12 weeks	[138, 165, 166, 170]
	PF	In vitro/in vivo	● Intraperitoneal; 50 mg/kg/day; 8 weeks	[163, 169, 177]
*Artemisia annua* Herba	Artesunate	MRL/lpr mice	● Intraperitoneal; 50 mg/kg/day; 8 weeks	[185, 186]
	DHA	Cellular/animal models	● Intraperitoneal; 50 mg/kg/day; 4–8 weeks	[183, 187–190, 192]
	Artemisinin	Preclinical models	● Oral gavage; 100 mg/kg/day; 8 weeks	[180, 181, 183, 184, 193, 194]

**Table 3:** Mechanistic summary of low evidence TCM interventions targeting the gut–kidney axis in LN

Active Ingredient	LN therapeutic mechanisms	Gut microbiota-related mechanisms	Evidence level	References
Curcumin	➣ Inhibits NF-κB/MAPK/PI3K-AKT ➣ Inhibits NLRP3 inflammasome ➣ ↓ Renal lymphocyte infiltration ➣ ↓ Anti-dsDNA	♦ ↑ *Lactobacillus/Bifidobacterium/Akkermansia* ♦ ↓ *Enterobacteriaceae*, ↓ F/B ratio ♦ ↑ Tight junction proteins ♦ Metabolized by gut microbiota	Low	[115–121, 126–129]
Triptolide	➣ Inhibits JAK/STAT1/CXCL10 ➣ Inhibits TLR4/NF-κB ➣ ↓ TNF-α/IL-6/IL-17 ➣ ↓ Proteinuria	♦ Restores microbial homeostasis ♦ Strengthens intestinal barrier ♦ Metabolism/toxicity influenced by gut microbiota	Low	[134, 135, 137, 139, 149]
Celastrol	➣ ↑ CD4^+^Foxp3^+^ Tregs ➣ ↓ Pro-inflammatory factors ➣ Restores immune tolerance	♦ ↑ Microbial diversity ♦ ↑ Beneficial metabolites ♦ Efficacy depends on intact microbiota	Low	[141, 143–148]
Tripterygium Glycosides	➣ ↑ Remission rate when combined with glucocorticoids ➣ ↑ Serum C3/C4	♦ Ameliorates dysbiosis in ankylosing spondylitis ♦ Modulates microbiota-metabolite-host axis	Low	[140, 151]
Baicalin	➣ Inhibits mTOR pathway ➣ Regulates Tfh/Tfr balance ➣ ↓ Autoantibodies	♦ ↑ SCFA-producing bacteria ♦ Metabolized by gut microbiota to baicalein ♦ Gut‒liver/gut‒brain axis effects	Low	[101, 153, 156–160]
Baicalein	➣ Activates Nrf2/HO-1 pathway ➣ Inhibits NLRP3 inflammasome ➣ ↓ Oxidative stress/inflammation	♦ Reshapes microbial community structure ♦ Effects abolished by antibiotic depletion	Low	[154, 155]
TGP	➣ Regulates T/B cell activation ➣ ↑ Treg function ➣ Inhibits NF-κB/JAK2/STAT3 ➣ Inhibits podocyte PANoptosis	♦ Corrects dysbiosis in autoimmune models ♦ ↑ *Lactobacillus* ♦ Inhibits Lyn/Snail pathway in gut epithelium	Low	[138, 165, 166, 170]
PF	➣ ↑ mTNF-α in M2 macrophages ➣ ↑ Treg expansion via TNFR2 ➣ Corrects Th17/Treg imbalance	♦ Metabolized by gut microbiota to benzoic acid ♦ Modulates indole-3-lactate production ♦ Regulates intestinal epithelial autophagy	Low	[163, 169, 177]
Artesunate	➣ Inhibits Tfh differentiation ➣ Inhibits JAK2-STAT3 pathway ➣ ↓ MCP-1, BAFF, autoantibodies	♦ ↑ *Akkermansia* in metabolic disorder models ♦ Reverses high-fat diet-induced dysbiosis	Low	[185, 186]
DHA	➣ Inhibits TLR4/IRF3 pathway ➣ ↓ Type I interferons ➣ Targets ITK/BTK in Tfh/B cells ➣ ↓ Germinal center B cells	♦ Restores α-diversity in colitis models ♦ ↑ *Bacteroidetes/Actinobacteria/Bifidobacterium* ♦ ↓ *Proteobacteria* ♦ Repairs intestinal barrier	Low	[183, 187–190, 192]
Artemisinin	➣ Inhibits NF-κB/TGF-β1 pathway ➣ ↓ TNF-α/IL-6 ➣ ↓ Renal inflammation/fibrosis	♦ Modulates key bacterial genera (*Anaerotruncus*) ♦ Subtle but functionally relevant shifts	Low	[180, 181, 183, 184, 193, 194]

Evidence levels are defined as follows: Low evidence (extrapolated): efficacy confirmed in LN models, but gut–kidney axis evidence derived from other disease models; causality in LN requires further validation

*F/B ratio* Firmicutes/Bacteroidetes ratio, *TGP* total glucosides of paeony, *PF* paeoniflorin, *DHA* dihydroartemisinin, *ITK* Interleukin-2-inducible T cell kinase, *BTK* Bruton's tyrosine kinase

Curcumin, derived from *Curcuma longa* Rhizoma, exerts immunomodulatory and renoprotective effects in LN by inhibiting certain signaling pathways (NF-κB, MAPK, and AKT), reducing renal T-cell and B-cell infiltration, lowering anti-dsDNA antibodies, and modulating splenic lymphocyte function [112–117]. Curcumin also suppresses neutrophil chemotaxis, inhibits NLRP3 inflammasome activation, alleviates podocyte injury, and improves renal fibrosis [117–120]. Preliminary clinical studies have suggested that oral curcumin improves proteinuria and blood pressure in LN patients and that it has a favorable safety profile [121–123].

Curcumin bidirectionally interacts with the gut microbiota. Gut microbes metabolize curcumin into more active derivatives, such as tetrahydrocurcumin, enhancing its bioavailability [36, 124, 125]. In turn, curcumin increases the abundance of beneficial bacteria (including *Lactobacillus*, *Bifidobacterium*, and *Akkermansia)*, suppresses the abundance of pathogens (such as *Enterobacteriaceae)*, and reduces the *Firmicutes* to *Bacteroidetes* ratio [36, 126–130]. Curcumin also enhances intestinal barrier function, inhibits TLR4 and NF-κB signaling, reduces LPS translocation, and modulates immunity via the gut–kidney axis [126, 131, 132].

(2) Triptolide, celastrol, and tripterygium glycosides

Triptolide, celastrol, and Tripterygium glycosides, which are *Tripterygium wilfordii* Radix constituents, demonstrate multitarget immunomodulation in LN [133, 134]. Triptolide inhibits JAK, STAT1, CXCL10, TLR4, and NF-κB signaling, reducing renal lymphocyte infiltration and proteinuria [134–139]. Tripterygium glycosides combined with glucocorticoids improve clinical remission rates and complement levels [140]. Celastrol expands Treg cells and suppresses Th17 responses [133, 141, 142].

These compounds also regulate the gut microbiota in other disease models. Celastrol restores microbiota diversity and exerts anti-inflammatory effects through microbiota-dependent mechanisms in patients with colitis and obesity [143–148]. Triptolide increases probiotics and suppresses pathogens in IBD models, with hepatotoxicity linked to microbiota metabolism [149]. Tripterygium glycosides reshape microbial structure and enhance intestinal barrier function in ankylosing spondylitis [150, 151].

(3) Baicalin and baicalein

Baicalin and baicalein, which are *Scutellaria baicalensis* Radix constituents, show therapeutic efficacy in LN [152]. Baicalin inhibits mTOR activity, regulates the Tfh/Tfr balance, and alleviates renal damage in MRL/lpr mice [153]. Baicalein activates Nrf2 and HO-1 signaling and inhibits the NLRP3 inflammasome in pristane-induced lupus models [154].

Both compounds target the gut microbiota in other disease models. Baicalein reshapes the microbiota, increases SCFA-producing bacteria, and improves lung barrier function via the gut–lung axis in ARDS; these effects are abolished by microbiota clearance [155]. Baicalin regulates microbiota composition, promotes beneficial metabolites, and strengthens the gut barrier in various disease models including heat stress, metabolic disorders, and atherosclerosis with depression [21, 101, 156–160].

(4) Total glucosides of paeony (TGP) and paeoniflorin (PF)

TGP exerts synergistic multicomponent effects in LN through immune modulation, inflammation suppression, and renal protection [161–163]. TGP regulates T- and B-cell activation, promotes anti-inflammatory macrophage polarization, and inhibits podocyte PANoptosis [138, 164–166].

PF, the main active component, replicates the effects of TGP by correcting Th17 and Treg imbalance, suppressing NF-κB and JAK signaling, and mitigating podocyte injury [163, 167–169].

Both TGP and PF modulate the gut microbiota. In autoimmune and inflammatory disease models, including rheumatoid arthritis, Sjögren's syndrome, and inflammatory bowel disease, both TGP and PF increase the abundance of beneficial bacteria (such as *Lactobacillus*), restore alpha diversity, and enhance intestinal barrier function via upregulation of ZO-1 and occludin expression [170–178]. PF is metabolized by the gut microbiota to benzoic acid and inhibits indole-3-lactic acid synthesis, regulating intestinal epithelial autophagy [177].

(5) Artemisinin, artesunate and DHA

Artemisinin and its derivatives have therapeutic potential in treating LN [179–184]. Artesunate, a water-soluble derivative, reduces renal IL-6 levels, suppresses Tfh differentiation via JAK2 and STAT3, and decreases autoantibody production [181, 185, 186].

DHA, the primary active metabolite, inhibits TLR4 and NF-κB, suppresses type I interferon, targets the interleukin-2-inducible T cell kinase (ITK) and Bruton's tyrosine kinase (BTK) pathways in Tfh and B cells, and activates Nrf2 and HO-1 in myeloid-derived suppressor cells [181, 183, 187, 188]. Artemisinin inhibits renal NF-κB and TGF-β1, reducing inflammation and fibrosis [181].

These compounds regulate the gut microbiota in animal models. In colitis and myasthenia gravis models, DHA restores alpha diversity, increases the abundance of beneficial bacteria (including *Bacteroidetes*, *Actinobacteria*, and *Bifidobacterium)*, and reduces the abundance of *Proteobacteria* [189–192]. Artesunate increases the abundance of *Akkermansia* in metabolic disorders, while artemisinin modulates key genera, such as *Anaerotruncus,* involved in metabolic–immune crosstalk [193, 194].

## Conclusions

**Fig. 7:**
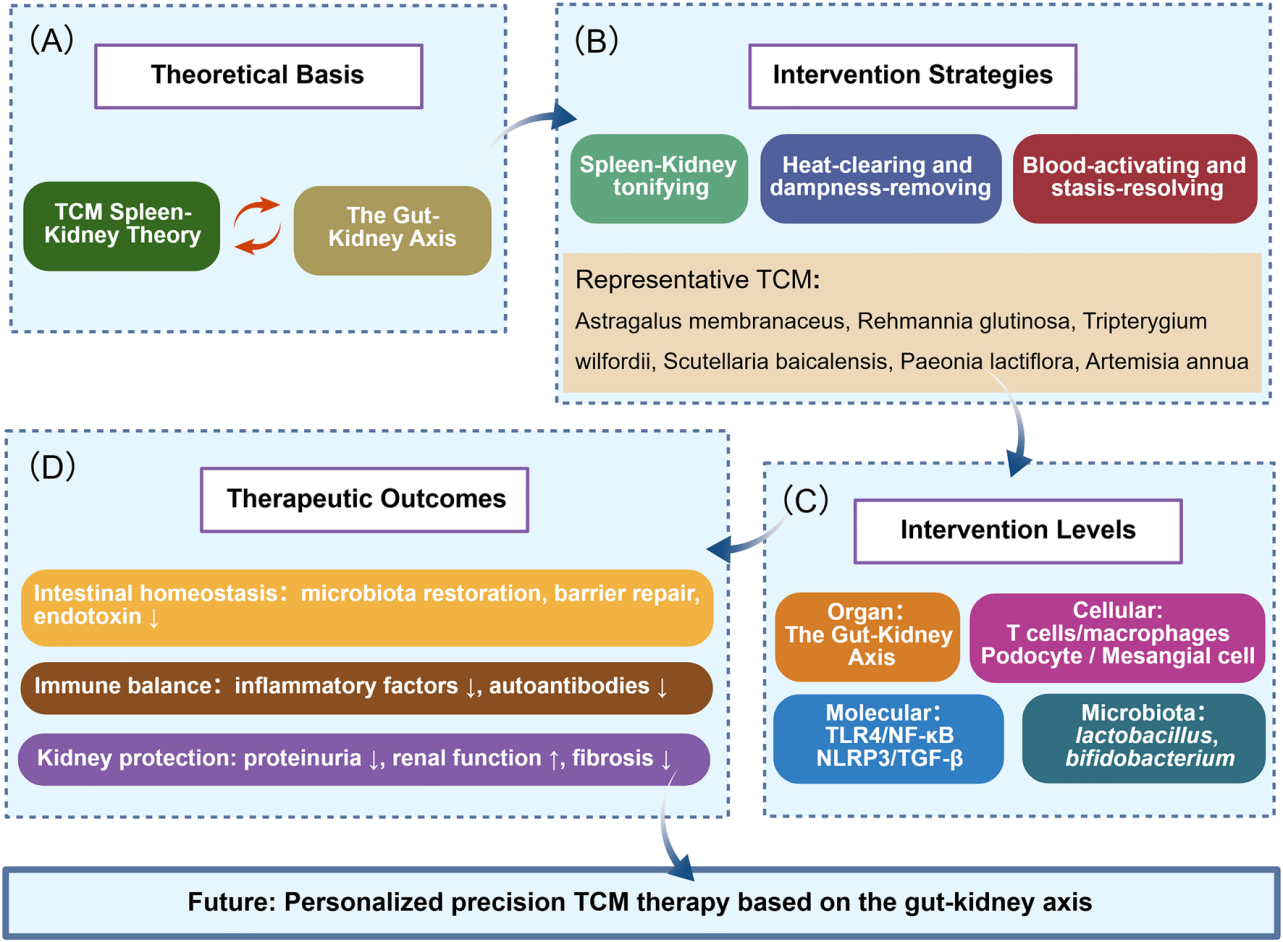
TCM therapeutic strategies targeting the gut–kidney axis in LN. **A** Theoretical basis: the TCM spleen–kidney correlation theory aligns with the modern gut–kidney axis concept. **B** Intervention strategies: representative therapeutic approaches and corresponding herbs. **C** Target levels: TCM acts at four levels—organs, cells, microbiota, and molecules. **D** Therapeutic outcomes: restoration of gut homeostasis, re-establishment of immune balance, and renal protection. This figure was created with BioGDP [13]

TCM shows promise for LN treatment by targeting the gut–kidney axis. This review links TCM syndromes to specific molecular pathways, including how spleen deficiency is related to intestinal barrier dysfunction and how dampness-heat is correlated with systemic inflammation [106]. Various TCM interventions, from single compounds to complex formulas, improve LN by reshaping the gut microbiota, repairing the intestinal barrier, and reducing inflammation (Fig. 7).

However, several critical limitations in the current evidence base must be acknowledged. First, the causal role of the gut microbiota in mediating TCM efficacy has been rigorously established for only a few interventions, most notably JP. For the majority of TCM interventions reviewed, including glycyrrhizic acid, Qinghao-Biejia, and various active compounds, the evidence remains largely correlational. Most studies have observed only concurrent changes in the gut microbiota and disease phenotypes following TCM treatment, without the use of germ-free animals, FMT, or antibiotic depletion experiments to establish causality. Second, the molecular targets of TCM interventions remain poorly characterized. Current research focuses primarily on phenotypic observations, and the specific receptors or signaling pathways through which TCM components affect the gut–kidney axis have not been systematically identified. Third, significant individual heterogeneity in gut microbiota composition may lead to variable therapeutic responses, yet few studies have investigated the relationship between baseline microbiota characteristics and treatment outcomes in LN patients [195].

Future studies should address these limitations through the following approaches. First, for promising agents such as curcumin and baicalin, researchers should perform FMT experiments in LN mouse models to confirm whether the transfer of gut bacteria from treated mice has therapeutic effects. Second, gene knockout mice or specific inhibitors should be used to identify host pathways required for TCM efficacy, rather than relying solely on observational microbiota profiling. Third, clinical trials should collect baseline stool samples to test whether initial microbiota composition predicts patient response, enabling personalized treatment. Fourth, longitudinal multiomics tracking of both bacteria and metabolites can reveal which microbial functions actually drive treatment response.

In summary, TCM offers practical therapeutic options for LN through gut–kidney axis modulation. Moving forward, the field needs fewer descriptive sequencing studies and more mechanistic experiments that demonstrate causation, identify targets, and account for individual patient differences. These steps help translate TCM from traditional practice into evidence-based therapies with clear indications for specific patient subgroups [196].

## Data Availability

No datasets were generated or analysed during the current study.

## Abbreviations

AS: *Astragalus membranaceus* and *Salvia miltiorrhiza*
BPYSF: Bupi yishen formula
BTK: Bruton's tyrosine kinase
CKD: Chronic kidney disease
DHA: Dihydroartemisinin
FMT: Fecal microbiota transplantation
HDAC: Histone deacetylase
HKC: Huangkui capsule
ITK: Interleukin-2-inducible T cell kinase
JP: Jieduquyuziyin prescription
LN: Lupus nephritis
LPS: Lipopolysaccharide
PAMP: Pathogen-associated molecular pattern
PF: Paeoniflorin
PRR: Pattern recognition receptor
RTK-PKCα: Receptor tyrosine kinase-protein kinase C alpha
SCFA: Short-chain fatty acid
SLE: Systemic lupus erythematosus
TCM: Traditional chinese medicine
TGP: Total glucosides of paeony
TMAO: Trimethylamine N-oxide
YSHS: Yi-Shen-Hua-Shi granule
